# Evaluating the Clinical Effectiveness and Patient Experience of a Large Language Model–Based Digital Tool for Home-Based Blood Pressure Management: Mixed Methods Study

**DOI:** 10.2196/68361

**Published:** 2025-11-03

**Authors:** Alan Jelic, Igor Sesto, Luka Rotkvic, Luka Pavlovic, Nikola Erceg, Nina Sesto, Zeljko Kraljevic, Joshua Au Yeung, Amos Folarin, Richard Dobson, Petroula Laiou

**Affiliations:** 1 Magdalena Clinic for Cardiovascular Diseases, Zagreb, Croatia; 2 Megi Health Ltd, London, United Kingdom; 3 Department of Psychology, Faculty of Humanities and Social Sciences, University of Zagreb, Zagreb, Croatia; 4 King's College London, 16 De Crespigny Park, London, SE5 8AB, United Kingdom, 44 020 7848 0002; 5 King's College Hospital NHS Foundation Trust, London, United Kingdom

**Keywords:** hypertension, large language models, LLMs, digital tools, artificial intelligence, AI

## Abstract

**Background:**

Hypertension, one of the most common cardiovascular conditions worldwide, necessitates comprehensive management due to its association with multiple health risks. Effective control often involves lifestyle changes and continuous monitoring, yet many individuals struggle to adhere to traditional management approaches. Digital health tools are emerging as promising alternatives, offering remote monitoring and real-time support. This study focuses on evaluating a digital tool specifically designed for hypertension management, analyzing its effectiveness, and gathering user perspectives on its functionality and impact.

**Objective:**

The primary objective of this study is to assess the effectiveness of a digital health tool in managing hypertension. Additionally, the study aims to understand user experiences and satisfaction levels to gauge the tool’s acceptance and potential for long-term use. By analyzing data from a large cohort, we seek to determine whether the tool can contribute to meaningful reductions in blood pressure and support sustained engagement over time.

**Methods:**

The study includes a cohort of 5136 participants who used the digital hypertension management tool. This tool provides continuous blood pressure monitoring, real-time feedback, and personalized health recommendations, which are crucial for tailored intervention. Participants recorded their blood pressure values over time, and we tracked retention rates to measure adherence. An online survey was administered to gather user feedback, focusing on ease of use, satisfaction levels, and perceived health benefits.

**Results:**

Our analysis indicates a significant reduction in blood pressure values among users, with a positive correlation observed between the duration of use and the extent of blood pressure reduction. We performed a 1-sided Wilcoxon Rank Sum test to compare systolic blood pressure values in the first and last biweekly use intervals, and descriptive statistics were used to assess survey responses. High retention rates were observed, with 2583 (50.3%) participants using the tool after 1 year. Survey responses revealed high satisfaction, with users highlighting the tool’s ease of use and noting reduced anxiety related to blood pressure management. These results suggest that users found the digital tool both effective and convenient.

**Conclusions:**

This study demonstrates the potential benefits of digital health tools in managing hypertension, emphasizing their ability to engage users over long periods and support blood pressure reduction. The high satisfaction rates and positive user feedback underscore the importance of user-centered design in creating effective health interventions. Overall, the findings suggest that digital tools, when designed with a focus on user experience, could be a valuable component in hypertension management strategies, complementing traditional health care approaches.

## Introduction

### Background

According to the American Heart Association, high blood pressure, called hypertension, is defined as systolic blood pressure (SBP) ≥130 mm Hg or diastolic blood pressure ≥80 mm Hg that remains elevated across time [1]. Hypertension affects over 1.2 billion people worldwide [2] and, in the last decades, the number of people with hypertension has doubled [3]. Hypertension is a major risk factor for cardiovascular diseases such as heart attack and heart failure, stroke, and chronic kidney disease [4]. Hence, effective management of hypertension is a key factor in reducing morbidity and mortality associated with cardiovascular complications.

Traditional approaches to hypertension management usually require regular visits to health care facilities for blood pressure monitoring and consultations. Such approaches can be burdensome for patients and place a strain on health care systems. Moreover, patients usually have difficulty with medication adherence and struggle to make lifestyle changes, with half of the patients stopping their treatment within a year [5], making this a complex and multifactorial challenge. These factors result in poor blood pressure control, leading to increased health risks, complications, mortality, and hospital admissions. To address these health issues, there is a growing interest in leveraging artificial intelligence (AI) and digital health technologies (eg, mobile apps and smartwatches) to facilitate home-based hypertension management. Such technologies offer long-term blood pressure monitoring, send reminders for the medication intake, provide educational content to support lifestyle changes, and provide personalized health advice.

Recent trials have demonstrated that digital applications can significantly lower baseline 24-hour ambulatory, home, and office SBP compared with the control group [6,7]. Since 2022, there has been an explosion of interest in large language models (LLMs) for their conversational ability and their potential as clinical AI chatbots. Current LLMs built on the transformer architecture have demonstrated comprehensive medical knowledge [8], human-level empathy [9], and comparable bedside manner [10] and medical safety to clinicians. Implementation of an LLM-enhanced digital solution may improve user interaction and efficacy.

While the clinical benefits of long-term blood pressure monitoring with digital technology are well documented [11], it is of paramount importance to also understand the effectiveness for blood pressure reduction as well as perspectives of users for such digital tools. Investigating users’ perspectives for digital home–based blood pressure monitoring can contribute to the optimization and improvement of digital tools for hypertension as well as to ensure that such digital tools fulfill the needs and preferences of the users [12].

### Objectives

In this work, we analyze the effectiveness for blood pressure reduction, user retention, and satisfaction with an innovative digital health tool that operates as a chatbot called Megi. Megi actively communicates with patients on a daily basis, reminds them to monitor their blood pressure, submits other relevant biometric data, takes their medications, and enhances their lifestyle. Specifically, we analyze the retention and engagement of 5136 Megi users and investigate whether they can achieve blood pressure reduction. In addition, we present the results of an online survey that quantifies users’ perspectives about Megi. The insights obtained from this study can aid in the design, development, and effectiveness of digital tools for hypertension and enhance users’ satisfaction.

## Methods

### Study Design

We conducted an observational cohort study with 5136 Megi users who used the platform between June 2022 and the end of July 2024 and fully completed the onboarding process. A user was considered fully onboarded once they accepted the consent; entered their first name, last name, and date of birth; and set the blood pressure measurement reminders. These users were not recruited through traditional methods; rather, they joined the platform organically through clinical referrals, health campaigns, or personal interest in self-managing hypertension. First, we analyzed user engagement and retention on the platform across the whole user base to assess the platform’s overall success in retaining patients engaged and adherent with at-home blood pressure monitoring. We define retention rate as the percentage of users who continued to use Megi beyond specific time intervals after their initial use of the platform. In this study, we considered the intervals of 3, 6, 9, 12, and 24 months. Accordingly, users were grouped into cohorts based on the duration of their use of Megi. These durations correspond to the specified retention periods. We analyzed the retention based on two types of events: (1) blood pressure reporting and (2) any interaction with Megi.

Subsequently, we randomly selected a cohort of active users that had been using Megi for at least 3 months to evaluate their perspectives about Megi. The randomly selected users had to fill out an extensive online survey that consisted of 20 questions, which were designed to (1) evaluate the usefulness of Megi in improving self-management of hypertension, (2) assess their satisfaction, (3) describe their willingness to use Megi, and (4) assess their behavioral response to Megi.

### Ethical Considerations

The survey was conducted in June 2023, and all participants gave written informed consent that they agreed to participate in the survey. Data were stored on a secure cloud platform that adheres to protected health information management standards. The study was approved by the Magdalena University Hospital Research Ethics Committee (195/Inf-701/22).

### Digital Tool for the Management of Hypertension

All study participants used Megi, an AI-based digital health tool for hypertension management. Megi is a chatbot equipped with a specialized transformer-based AI model and is accessed via messaging apps such as WhatsApp and Viber. The chat is built on LLMs from the GPT family (GPT-3.5, GPT-4, and GPT-4o [OpenAI]). To date, various versions of OpenAI’s models have been used. These models are accessed via the Microsoft Azure OpenAI Service. Via this chatbot and through images, Megi gathers on a daily basis multimodal behavioral and biometric data from users that range from blood pressure and heart rate measurements to mood and stress levels. In addition, it provides personalized advice for lifestyle changes. On a daily basis, users receive reminders to measure their blood pressure and take their medications. All data are stored in secured cloud platforms, and clinicians have 24-7 access to all data via an online platform. Visual examples of the Megi platform and further information about Megi can be found on the Megi website [13].

### Survey Questions

The online survey consisted of 19 questions. There were 3 questions that collected information about the users’ age, sex, and employment status. In addition, there was 1 question that evaluated the impact of hypertension on the users’ daily life. The survey had 3 questions that assessed the usefulness of the technology in improving self-management of hypertension (ie, “I understand why it is important to measure your blood pressure daily”; “I would often forget to measure my blood pressure if Megi didn’t remind me”; and “With Megi, I will develop the habit of regular blood pressure measurement and monitoring”). Moreover, there were 3 questions that evaluated the users’ satisfaction (ie, “I can see the benefits of using Megi”; “I have confidence in Megi”; and “I would continue to use Megi even if my doctor did not access my data”). The willingness to use the digital tool was evaluated by 4 questions (ie, “I would definitely recommend Megi to my friends”; “Anyone who cares about their health should use Megi”; “For the sake of myself and my loved ones, it is important for me to take care of my health”; and “My loved ones and friends would encourage me to use Megi”), while the behavioral response of the users was also assessed by 4 questions (ie, “Since using Megi, I feel safer and more relaxed”; “I feel happy when I receive messages from Megi”; “Megi makes me feel less lonely”; and “I wouldn’t want to lose access to Megi”). Finally, the survey had 1 question that asked users to provide the unmet needs of the digital tool in free text. The answers were provided either in a 5-point Likert scale (1: I strongly disagree, 2: disagree, 3: neither agree nor disagree, 4: agree, and 5: strongly agree) or in free text.

### Statistical Analysis

We analyzed the blood pressure values of all users in the first 2 weeks after their enrollment in the Megi platform and in the last 2 weeks before they stopped reporting their blood pressure values. For every user, we computed the average blood pressure values in the first and last biweekly segment. To quantify the statistical differences between the first and last biweekly segments, we applied a 1-sided nonparametric Wilcoxon Rank Sum test. Results were considered statistically significant if their *P* value was less than .05. In addition, we performed a descriptive analysis to assess the results of the online survey. Informative quotes were provided for the answers that were given in free text. All analysis was performed in MATLAB R2022a.

## Results

### User Retention

**Figure 1.**
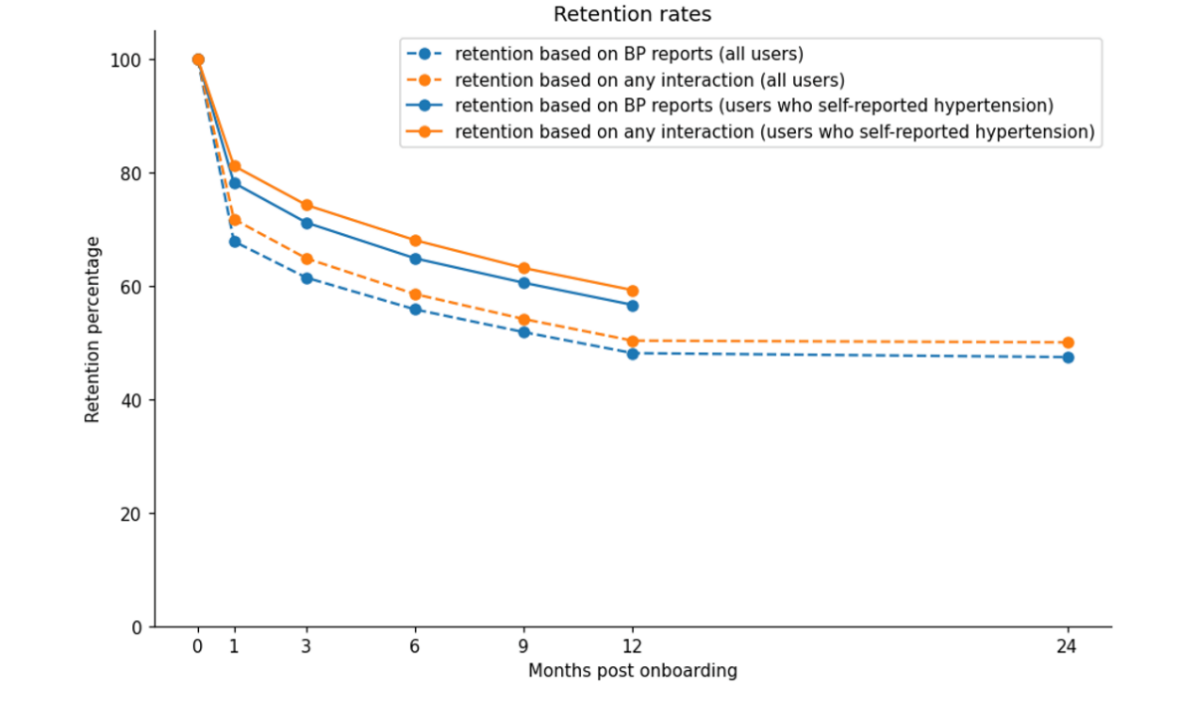
User retention (N=5136) across months after onboarding based on blood pressure (BP) reporting (orange) and any interaction with Megi (blue). Dotted lines correspond to all users, while solid lines correspond to users who self-reported that they had hypertension upon their enrollment (this feature was implemented in the second year).

We first analyzed the retention of 5136 Megi users (n=2399, 46.71% female; n=2733, 53.21% male; n=4, 0.001% other; median age 59, range 17‐95 y). Retention was quantified based on (1) blood pressure reporting and (2) any interaction with Megi. Figure 1 demonstrates that the retention rates after one year of using Megi were 50.3% for users based on all their interactions with Megi (eg, updating weight data, reacting to therapy reminders, and submitting feedback) and 48.1% for users based on their blood pressure measurement reports. The retention rates were even higher, that is, 60.5 and 63.1% respectively, for users who self-reported being hypertensive. Interestingly, user retention remained stable even after 2 years of using Megi.

### Blood Pressure Analysis

**Figure 2.**
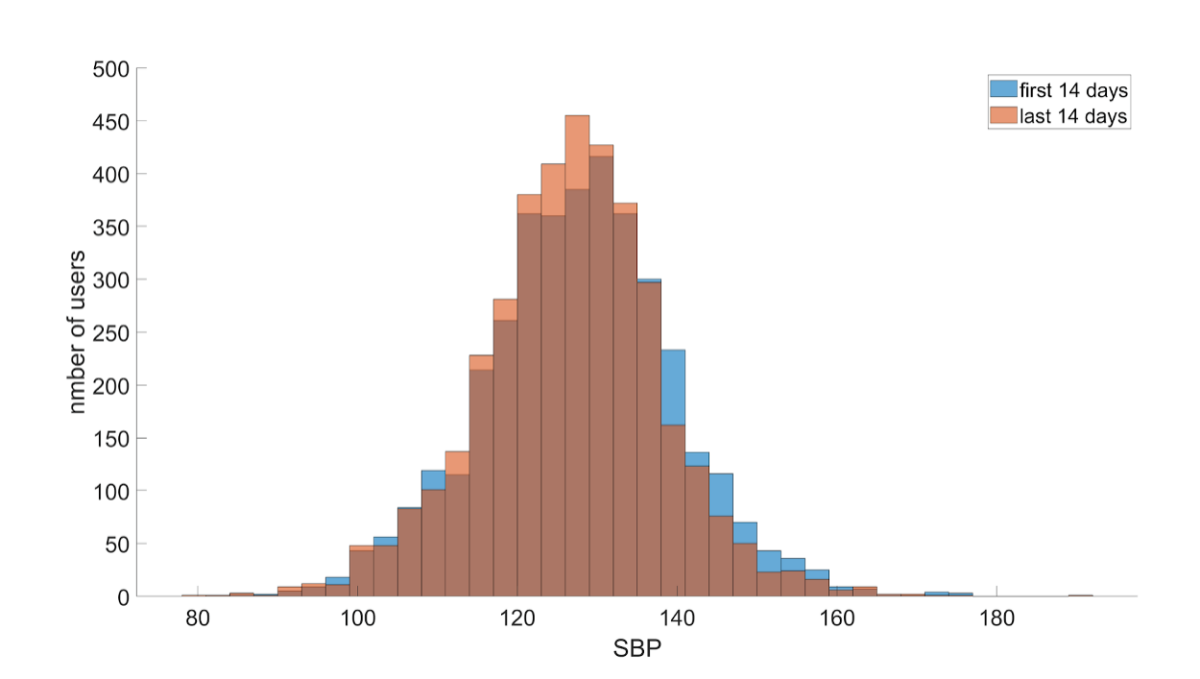
Distributions of the average systolic blood pressure (SBP) measurements during the first biweekly segment after study enrollment (blue) and last biweekly segment (orange) before users paused reporting blood pressure values.

In total, 4736 (92.21%) of 5136 users reported their blood pressure values. We first sought to uncover whether there was a statistically significant difference in the average SBP measurements between the first and last biweekly segment. Figure 2 illustrates the distributions of the average SBP values of all users for the first biweekly segment after their enrollment and the last biweekly segment for which users reported their blood pressure values. We applied a 1-sided Wilcoxon rank sum test and found that the SBP values in the last biweekly segment were statistically significantly reduced compared to the first biweekly interval (*P*<.001).

**Figure 3.**
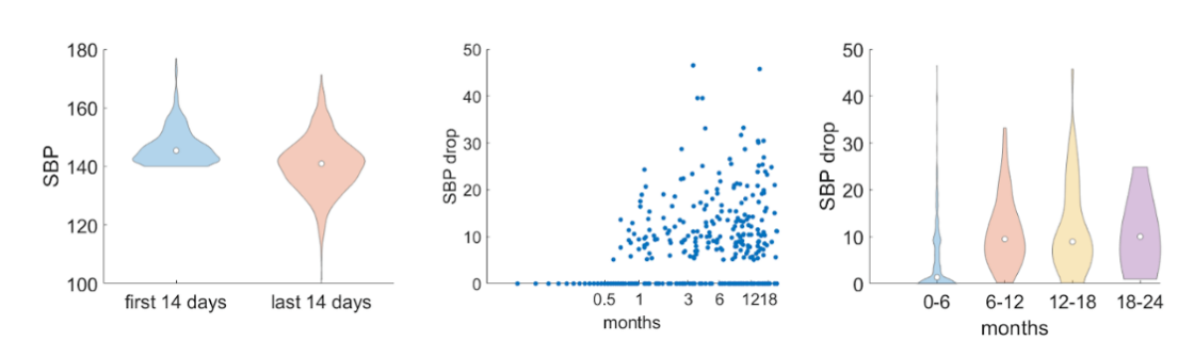
Left: distributions of systolic blood pressure (SBP) measurements for users whose average SBP value was at least 140 mm Hg during the first weekly biweekly interval after enrollment (blue). The orange violin plot depicts the average SBP values during the last biweekly interval for which users reported their blood pressure values. Center: SBP drop across the time (in mo) for which users report their BP measurements. Each dot corresponds to a different user. Right: distributions of the SBP drop across different time intervals (in mo) for which users report the blood pressure measurements.

Afterward, we wanted to explore whether such SBP differences were stronger for users whose average SBP values were at least 140 mmHg in the first biweekly segment (Figure 3). We saw that such differences between the first and last biweekly interval were much stronger (*P*=1.7×10^−9^). We also examined the correlation between the SBP drop (ie, the difference of the average SBP during the first biweekly interval and the last biweekly interval) and the period for which users report their blood pressure values. The Spearman correlation was 0.53, and it was statistically significant (*P*=4.5^−32^), which means that larger SBP reduction is positively correlated with longer use of Megi. Figure 3 illustrates the SBP drop across the months for which users reported their blood pressure values. The maximum duration in blood pressure reporting was 24 months, and we observed that there was an increasing trend of the SBP drop across time.

**Figure 4.**
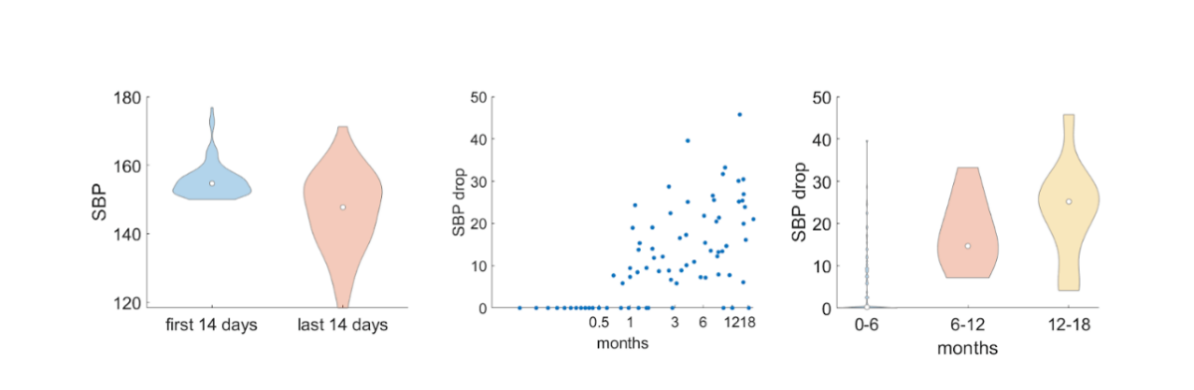
Users whose average systolic blood pressure (SBP) was at least 150 mm Hg during the first biweekly interval after their enrollment.

We performed a similar analysis for the users whose average SBP was at least 150 mm Hg in the first biweekly segment (Figure 4). For users with such high blood pressure, the differences between the first and last biweekly interval were also strong (*P* value=1.7×10^−9^) and the correlation between the SBP drop and the duration for which users report their blood pressure measurements was even stronger (ρ=0.75). Figure 4 also illustrates a clear increase in the SBP drop across time.

### Online Survey Analysis: Dataset Characteristics

A total of 129 people clicked on a link to access the survey (June 2023). The total number of analyzed participants is 125 (96.9%; n=3, 2.33% participants were excluded from the analysis due to the presence of missing values). Summary demographics regarding participants’ age, sex, and employment status are provided in Table 1 and Figure 1. Out of the 125 participants, 55 (44%) were female, and the age of all survey participants ranged from 23 to 82 (mean 60, SD 11.6) years.

At first, study participants were asked whether high blood pressure has a significant impact on their life. The vast majority of the participants strongly agreed (n=58/125, 47%) or agreed (36/125, 29%) while just 23 of 125 (19%) strongly disagreed or disagreed.

**Table 1.** Dataset characteristics (N=125).

Characteristics	Users, n ( %)
Age group (y)	
20-29	2 (1.6)
30-39	3 (2.4)
40-49	18 (14.4)
50-59	27 (21.6)
60-69	52 (41.6)
70-79	20 (16)
80-89	1 (0.8)
Sex	
Male	70 (44)
Female	55 (56)
Employment status	
Employed	54 (43.2)
Unemployed	5 (4)
Retired	64 (51.2)
Student	1 (0.8)

Participants were asked a series of questions to evaluate the usefulness of technology in managing their hypertension. The responses indicated a high level of perceived usefulness. Overall, 80% (100/125) of the participants agreed or strongly agreed that Megi helped them understand their hypertension better. Regarding the importance of blood pressure monitoring, 98% (122/125) found the technology useful for regularly monitoring their blood pressure. When the participants were asked whether they would forget to measure their blood pressure if Megi did not remind them, 80% (100/125) responded that they would. In addition, study participants felt that using Megi made them develop the habit of measuring their blood pressure regularly (118/125, 95% responded positively).

When participants answered questions that assessed their overall perception, confidence in the digital tool, and willingness to continue using it independently of their health care provider, again their overall satisfaction was very high. Specifically, regarding the statement “I can see the benefits of using Megi,” 98% (122/125) of the study participants strongly agreed or agreed. In addition, when participants were asked whether they had confidence in Megi, 92% (115/125) responded positively, while when they were asked whether they were willing to use Megi independently, more than 80% (100/125) agreed or strongly agreed.

Study participants were also assessed regarding their willingness to use or recommend Megi. Specifically, they asked whether they (1) would recommend Megi to their friends (112/125, 90% of the users responded positively) and (2) would agree with the following statements: “Anyone who cares about their health should use Megi” (100/125, 80% of the users strongly agreed or agreed); “For the sake of myself and my loved ones, it is important for me to take care of my health” (112/125, 90% of the users responded positively); and “My loved ones and friends would encourage me to use Megi” (more than 55% of the users responded positively).

At the end of the survey, participants had to answer questions regarding their behavioral response with the digital tool. They had to answer the following questions: “Since using Megi, I feel safer and more relaxed”; “I feel happy when I receive messages from Megi”; “I wouldn’t want to lose access to Megi”; and “Megi makes me feel less lonely.” Across all questions but the last, more than 80% (100/125) of the participants felt that Megi reduced their anxiety and made them feel safe.

### Unmet Needs

Study participants were also asked whether there is something that they would change about Megi. Answers were given in free text. Some users were asking for more personalized treatment (eg, “I think maybe she should adjust according to the specifics of individual patients. I can illustrate this with my own example. I have been hypertensive for about thirty years, I regularly measure my blood pressure, so I don’t think it’s necessary to report my condition every two weeks”; “I would like to determine more precisely what type of pressure I have...when it is really low and when it is high, and based on the measurements…”) or the possibility of clinical consultations with cardiologists (eg, “Maybe the availability of a cardiologist to guide me”; “If there were an opportunity to occasionally discuss the results with a doctor, I think I would take regular measurements more seriously”). On the basis of the qualitative feedback, several common themes emerged that can inform future improvements to Megi. These user-driven recommendations are summarized here—(1) personalization of reminders: allow users to adjust the frequency or timing of blood pressure measurement reminders based on their experience and needs; (2) flexible reporting options: give experienced users the option to reduce or customize the frequency of data entry, especially if their condition is stable; and (3) clinical support integration: offer optional access to health care professionals (eg, cardiologists or general practitioners) for periodic review or consultations.

## Discussion

### Principal Findings

The management of hypertension at home using digital apps presents a promising avenue for improving patient outcomes and alleviating the burden on health care systems. This study explored users’ perspectives on Megi, a digital tool designed to facilitate long-term home-based blood pressure monitoring, and demonstrated the effectiveness of Megi on blood pressure reduction.

Analyzing the activity of 5136 users, our results show very high engagement and adherence to blood pressure readings that remain stable even after one year of using Megi (Figure 1). Specifically, more than 50% of the users are active on the Megi digital health platform for 12 months, which demonstrates higher user retention when compared to previously reported industry standards. For example, consistent with other large-scale digital health studies, the notable Stanford-led MyHeart Counts study experienced substantial dropout rates; mean engagement with the app was only 4.1 days [14]. Statista research into the worldwide retention rate of mobile app installs reveals that, on the day of installation, the average retention rate across the 31 categories was 25.3% [15]. Worryingly, by day 30, this percentage drops to just 5.7%, and when it comes to the health and wellness industry specifically, it is even lower at 2.8% [15].

In addition, our analysis showed the effectiveness of Megi as a digital tool for hypertension management (Figures2^4^). We demonstrated that the average SBP of the last biweekly interval for which users reported their blood pressure values was statistically significantly smaller compared to the first biweekly segment after users’ enrollment (Figure 2). This difference was even stronger for users who had high (>140 mm Hg; Figure 3A) and very high SBP measurements (>150 mm Hg; Figure 4A) during the first biweekly segment. Furthermore, we showed that there is a positive correlation between the SBP drop and the duration for which users report their blood pressure values. This means that larger SBP reductions are associated with longer use of Megi (Figures 3, 4B, and 4C).

While our results demonstrate a significant association between longer Megi use and reductions in SBP, it is important to note that the observational nature of the study limits causal inference. Without a control group, we cannot fully exclude the impact of confounding variables such as concurrent lifestyle changes, improved medication adherence independent of Megi, or parallel support from health care providers. These factors may have contributed, in part, to the observed improvements. Future randomized controlled trials are needed to validate these findings and better isolate the direct effect of the digital tool on clinical outcomes.

Through an online survey, we evaluated users’ perspectives on the technology’s usefulness in improving self-management of hypertension, their satisfaction with the tool, their willingness to use it, and their behavioral responses to its implementation. The findings indicate a high level (ie, more than 90%) of user satisfaction with Megi. Participants strongly acknowledged the technology’s usefulness in enhancing their ability to manage hypertension independently (Figures3  4), as well as expressed their willingness to suggest Megi to other users. The tool’s integration into daily routines led to a noticeable reduction in anxiety and an increase in feelings of relaxation and happiness, suggesting a positive impact on mental well-being. The behavioral response data—particularly users reporting feeling safer, less anxious, and more connected—aligns with previous findings on the importance of emotional engagement in digital health interventions. Previous studies have shown that user adherence improves when digital tools provide empathic, human-like interaction, particularly in long-term chronic condition management [9,12]. Megi’s use of LLM-powered messaging may have contributed to these outcomes by delivering timely, personalized, and conversational support that mimics human dialog. This is consistent with recent research demonstrating that LLMs can convey human-like empathy and reassurance in health contexts [8,10]. Furthermore, the willingness of users to recommend Megi to friends reflects strong approval and confidence in its efficacy. Overall, the positive feedback from users highlights the potential of Megi as a valuable digital tool for hypertension management.

### Limitations

Despite the valuable insights, this study has limitations that must be acknowledged. First, its observational design limits the ability to draw causal conclusions. Second, the online survey relied on convenience sampling and included only users with at least 3 months of continuous use, excluding those with intermittent engagement. This may introduce selection bias and limit generalizability. In addition, the single ethnicity of the analyzed cohort limits the generalizability of the findings. Cultural attitudes toward technology, patient-clinician relationships, and the structure of local health care systems may influence user engagement, retention, and perceived value of digital tools. Moreover, the evaluation focused exclusively on one digital tool, that is, Megi, which may not provide a comprehensive understanding of the broader landscape of digital tools for hypertension management. These constraints may affect the generalizability of the findings and highlight the need for further research with more diverse and larger populations to validate and expand upon these results.

### Recommendations

In addition, we recommend that future research include more diverse populations to enhance the generalizability of results and evaluate clinically validated outcomes such as cardiovascular events and treatment modifications. Incorporating structured metrics for therapeutic compliance and integrating the platform with clinical workflows would further strengthen its clinical utility. In addition, prospective studies—particularly randomized controlled trials—are needed to establish causal relationships and assess the long-term impact of digital tools such as Megi on hypertension management and overall cardiovascular health.

### Conclusions

Our study demonstrated the effectiveness of a digital tool for hypertension management and highlighted the importance of understanding user views in the design and implementation of digital health interventions for hypertension management. In conclusion, the insights obtained from this study highlight the potential of digital apps to enhance hypertension management by providing continuous monitoring, real-time feedback, and personalized health recommendations.

## Abbreviations

AI: artificial intelligence
LLM: large language model
SBP: systolic blood pressure
